# Editorial of Special Issue “The Role of Vitamin D in Human Health and Diseases”

**DOI:** 10.3390/ijms23084283

**Published:** 2022-04-13

**Authors:** Francesca Silvagno, Loredana Bergandi

**Affiliations:** Department of Oncology, University of Torino, Via Santena 5 bis, 10126 Torino, Italy

Vitamin D has been described as a differentiative hormone, but this definition is reductive for a molecule targeting every tissue, produced in its active form by many kinds of cells and effective on the whole life of cells by different mechanisms, which lead to nuclear, non-genomic and mitochondrial effects. Furthermore, in addition to its well-known impact on several functions, such as bone remodeling, skin differentiation and the immune system, just to cite a few, many other tissues depend heavily on vitamin D for their health; therefore, the correlation between low levels of vitamin D and the onset of many diseases has been reported. In this Special Issue, we published five experimental studies and a compendium of five review articles covering different aspects about the role of vitamin D in health and diseases, not only with the intent to reveal new findings and to discuss the current literature, but also to stimulate the search for novel therapeutic strategies, exploiting the multiple pathways influenced by the hormone.

In this collection, four reviews investigated the correlations between vitamin D deficiency and diabetes [1], sleep disorders [2], rheumatic diseases [3] and COVID-19 [4]. Although the results of some clinical trials supplementing vitamin D are encouraging, the few discrepancies can be due to differences in therapy protocols, resistance to the hormone and, in general, can be explained by an individual sensitivity to its action. The heterogeneity of administration protocols in terms of duration and doses, and the need for personalized supplementation, are discussed in two review studies investigating rheumatic diseases [3] and COVID-19 [4]. Among the causes of variable sensitivity is surely to be mentioned the vitamin D receptor (VDR) polymorphism. Concerning FokI VDR polymorphism, the experimental omics approaches employed by Colombini et al. [5] revealed that many processes such as matrix remodeling, collagen metabolism, angiogenesis and inflammatory cytokines were modulated in the opposite way in homozygous FF intervertebral disc cells and in heterozygous Ff cells. Analysis of the biochemical pathways influenced by vitamin D can support a specific approach to enhance the efficacy of the hormone for each disease, based on individual characteristics. For example, the findings from Colombini et al. [5] are relevant for a clinical investigation aiming to assess the potential of the administration of vitamin D in patients affected by disc degeneration, both in terms of response to the treatment, and the effect of the hormone on matrix remodeling and on hypertrophy of the tissues involved.

The pleiotropic activity of vitamin D can be exploited through cotreatment with other molecules impacting on the same pathways. This is discussed in reviews from Albergamo et al. [4] and Segovia-Mendoza et al. [6]. The former article concluded that the efficacy of vitamin D could be increased by combining it with other molecules that affect the mechanisms underlying severe COVID-19, such as antioxidant molecules or ROS scavengers; alternatively, limiting the use of drugs that are eliminated by conjugation with glutathione, thus consuming antioxidant defenses, such as paracetamol, could reinforce the antioxidant effects of vitamin D. Furthermore, the acidification of the vesicles, which would block the viral exit from infected cells, could be increased by the combination of vitamin D and beta-glucans [4]. The latter study discussed preclinical and clinical studies concerning the combination of vitamin D with different oncological drugs, emphasizing its main therapeutic benefits. In addition to the combination with chemo/radiotherapy, the authors extensively described the mechanisms and efficacy of several associations, such as natural compounds, endocrine therapy, histone modifiers, kinase and histamine inhibitors, and immunomodulatory agents [6]. In addition to combined treatments, the activity of vitamin D could be enhanced by improved delivery. The study from Cataldi et al. [7] investigated the effect of conjugating the hormone with silver nanoparticles (AgNPs), which are often employed on skin due to their antibacterial, antiviral, antifungal, and anticancer properties. The authors found that vitamin D-AgNPs combination is essential for a quick and complete wound repair and that it involves the sphingomyelin pathway as the signal transduction in human keratinocytes.

Among the pathways analyzed in this collection, the antioxidant and anti-inflammatory effect of vitamin D has been found especially important in many diseases, such as COVID-19 [4], diabetes [1] and asthma [8]. Oral supplementation with vitamin D seems to play a protective role in balancing the oxidative stress associated with asthmatic inflammation. In fact, Adam-Bonci et al. found that vitamin D reduced the oxidative components and increased the antioxidant capacity of the serum and lung tissue in a mice model of induced acute asthmatic inflammation [8]. The impact of vitamin D on fat metabolism and the endocannabinoidome-gut microbiota axis is reported in the study from Abolghasemi et al. [9]. These authors reported that increasing vitamin D levels promoted a reduction in body weight gain and fat mass in both vehicle and in the atypical antipsychotic drug olanzapine-treated groups of female mice fed with a high fat/high sucrose diet, confirming the effect of vitamin D on obesity. Olanzapine significantly decreased the levels of Muribaculaceae and Bifidobacteriaceae only under conditions of vitamin D supplementation, suggesting that these taxa may also be obesogenic. The thyroid gland, central to controlling the metabolism of adipose and bone tissue, also proved to be a target tissue of vitamin D, as reported by Šošic-Jurjevic et al. [10]. In a model of orchidectomized middle-aged rats for male osteoporosis, this original study demonstrated that vitamin D treatment induced changes in thyroid functional morphology, indicating increased utilization of stored colloid and the release of thyroid hormones to maintain hormonal balance.

As a whole, this Special Issue contains an interesting combination of articles that present novel data on the effects of vitamin D on intervertebral disc, gut–brain axis, skin, asthma and thyroid, and discusses current knowledge, existing controversies, and new approaches related to the role of vitamin D deficiency and supplementation in several diseases.
